# Difficult cannulation during endoscopic retrograde cholangiopancreatography—needle-knife precut versus transpancreatic sphincterotomy on the basis of successful cannulation and adverse events

**DOI:** 10.1007/s00464-024-11429-y

**Published:** 2024-12-29

**Authors:** Arvid Gustafsson, Bobby Tingstedt, Greger Olsson

**Affiliations:** 1 Department of Research and Development and Department of Surgery, Central Hospital, Region Kronoberg, Strandvägen 8, 351 85 Växjö, Sweden; 2 Department of Clinical Sciences Lund, Surgery, Lund University and Department of Surgery, Skåne University Hospital, Lund, Sweden

**Keywords:** Endoscopic retrograde cholangiopancreatography, Adverse events, Precut sphincterotomy, Transpancreatic sphincterotomy, Needle-knife sphincterotomy

## Abstract

**Background:**

When cannulation is challenging during endoscopic retrograde cholangiopancreatography (ERCP), and the standard guidewire technique with sphincterotomy is unsuccessful, alternative cannulation techniques can be used to access the biliary tree. The purpose of this study was to compare the incidence of adverse events and cannulation success rates between transpancreatic sphincterotomy (TPS) and precut sphincterotomy (PCS).

**Methods:**

Data from the Swedish Registry for Gallstone Surgery and ERCP (GallRiks), collected from 2011 to 2022, were analyzed. A total of 105,303 ERCP procedures were recorded in GallRiks during the study period. After exclusions, the study population consisted of 47,486 ERCP procedures. Of these, 4547 received PCS and 3273 received TPS. The remaining 39,666 ERCP procedures with conventional sphincterotomy served as the control group. The primary endpoints were successful cannulation and adverse events within 30 days.

**Results:**

Successful cannulation was more frequent with the TPS technique than with the PCS technique (86.5% vs. 69.7%), but both groups had a lower cannulation rate than the control group (92.4%; OR-PCS 0.20, 95% CI 0.18–0.21; OR-TPS 0.58, 95% CI 0.52–0.64). The TPS group had a higher incidence of adverse events than the PCS group (24.1% vs. 18.8%) and both groups had a higher incidence of adverse events than the control group (15.5%; OR-PCS 1.25, 95% CI 1.15–1.36; OR-TPS 1.71, 95% CI 1.57–1.87). Adverse events for TPS were driven by a higher incidence of pancreatitis (10.5% vs. 6.4% vs. 4.5%; OR 2.53, 95% CI 2.23–2.86) and perforation (1.6% vs. 0.8% vs. 0.5%; OR 2.99, 95% CI 2.20–4.06) compared to both PCS and control.

**Conclusion:**

TPS is more successful at cannulation than PCS; however, this success comes at a higher cost in terms of adverse events, particularly pancreatitis and perforation.

**Graphical abstract:**

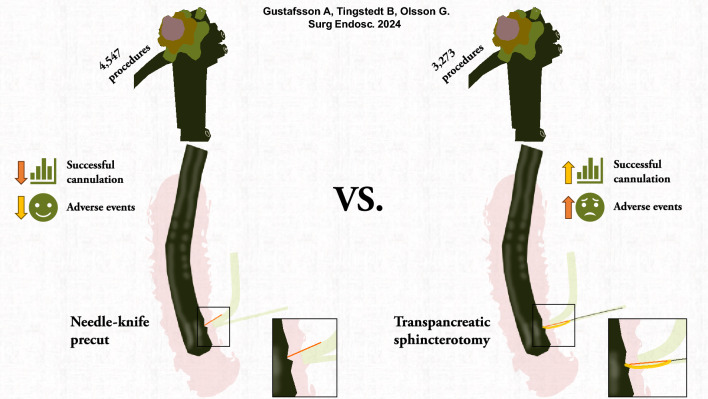

**Supplementary Information:**

The online version contains supplementary material available at 10.1007/s00464-024-11429-y.

The primary cause of adverse events following endoscopic cholangiopancreatography (ERCP) is difficulty in achieving successful deep cannulation of the common bile duct [1]. The incidence of adverse events after ERCP is 5–10%, with the most common being post-ERCP pancreatitis (PEP) [2, 3]. The risk of adverse events is increased with difficult cannulation, which includes repeated attempts of cannulation, manipulation of the papilla, and interference with the pancreatic duct [4]. The success rate of cannulation ranges from 80 to 95%, depending on the indication for the procedure and the experience of the endoscopist. While cannulating the biliary tree, the guidewire may be accidentally passed into the pancreatic duct. In this scenario, the double guidewire (DGW) technique can be used, which involves repeated attempts at cannulation with a second guidewire, while the first is in place in the pancreatic duct. Another option, or if this fails, is to perform a transpancreatic sphincterotomy (TPS). TPS involves using a sphincterotome on the guidewire accidentally placed in the pancreatic duct and cutting through the septum between the pancreatic duct and the common bile duct. This incision is usually made in the 11 or 12 o’clock direction. With the common bile duct opening visualized, cannulation can be established. A precut by needle-knife fistulotomy or sphincterotomy (PCS) can be used if no guidewire has been inserted into the pancreatic duct or if the common bile duct is clearly visible as a bulge in the mucosa. This technique uses a needle-knife papillotome and freehand cutting of the mucosa in an upward direction starting at the papillary orifice (sphincterotomy or papillotomy) or 3–5 mm above the orifice (fistulotomy) [5]. While these techniques may increase the likelihood of successful cannulation, they also carry a higher risk of adverse events. The majority of studies have indicated comparable adverse event rates between the two techniques [1, 6–14], similar cannulation rates [7, 11], and even a higher rate of successful cannulation with TPS [1, 6, 8–10, 12, 14]. However, persisting with the standard cannulation technique may also result in a higher risk of adverse events [1, 6, 15]. The sequence of techniques has also been widely debated, with DGW generally recommended before TPS [5, 16, 17]. However, some studies have argued that DGW may instead carry an increased risk of PEP compared to performing TPS directly after an unintentional pancreatic duct cannulation [15, 18, 19].

TPS and PCS are not strictly comparable because TPS is not possible if access to the pancreatic duct has not been established. However, the question of whether TPS should always be preferred to PCS has not been studied in larger cohorts. The aim of this study was to provide scientific evidence by comparing TPS and PCS cannulation techniques in terms of cannulation success and adverse event rates.

## Materials and methods

### Study design and population

**Fig. 1:**
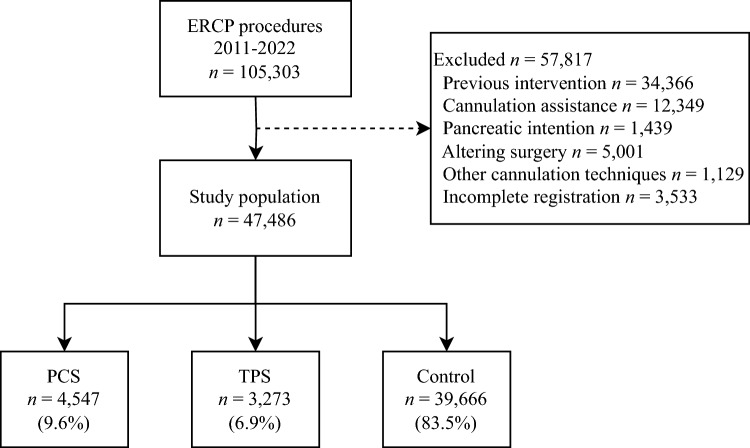
Flowchart illustrating excluded procedures and study cohort grouping. *PCS* precut sphincterotomy, *TPS* transpancreatic sphincterotomy

Using the Swedish Registry for Gallstone Surgery and ERCP (GallRiks), we conducted a population-based cohort study of ERCP procedures performed in Sweden from 2011 to 2022. Although the study design was retrospective, cohort data were prospectively collected in the registry. We utilized the Strengthening the Reporting of Observational Studies in Epidemiology (STROBE) checklist. Patients undergoing ERCP with either PCS, TPS, or a regular sphincterotomy technique were included. Patients with previous ERCP or sphincterotomy, cannulation assistance, pancreatic duct intention, altering surgery, other cannulation techniques, or incomplete registration were excluded (Fig. 1). A control group of procedures with regular sphincterotomy was established. Primary outcomes were successful cannulation and adverse events; specific adverse events were also analyzed.

### The GallRiks registry

The nationwide online GallRiks registry is supported by the Swedish National Board of Health and Welfare. The endoscopist performing the ERCP enters data directly into the registry variables during or shortly after the procedure. Several mandatory variables are entered, including both patient characteristics and variables that describe the procedure. After 30 days, an independent local coordinator reviews the relevant medical records for any adverse events, which are then recorded in the registry. The registry undergoes regular validation by independent observers at each participating center.

### Variables

*Precut sphincterotomy (PCS)* was defined as both the techniques of needle-knife sphincterotomy (more common) and needle-knife fistulotomy.

*Transpancreatic sphincterotomy (TPS)* was defined as using a guidewire inadvertently placed in the pancreatic duct and then using a sphincterotome to perform a sphincterotomy toward the bile duct orifice. A previous attempt using the double guidewire technique was allowed.

*Successful cannulation* was described as the ability to achieve deep contact with the bile duct visualized by the guidewire and fluoroscopic cholangiogram.

*Adverse events* were recorded within 30 days of ERCP and consist of a variable that summarizes all adverse events which also include intraprocedural complications. Specific adverse events were also recorded.

*Intraprocedural complications* were identified directly by the endoscopist during the ERCP procedure and include bleeding requiring intervention, perforation, or contrast extravasation.

*Age* was binned into four categories, represented as quartiles.

The *ASA (American Society of Anesthesiologists) classification* was divided into two groups: ASA 1–2 and ASA 3–4.

Whether a *duodenal diverticulum* was present and impacted the papilla during ERCP was only recorded for two types of diverticula: Boix type 1 and Boix types 2–3.

### Statistical analysis

Continuous variables are reported as means with standard deviations (SD), while categorical variables are presented as absolute frequencies with percentages. Missing data were assessed in all groups but excluded from the analysis due to low and even distribution. The threshold for statistical significance was established at a two-tailed *p* value of less than 0.05. Odds ratios (OR) and 95% confidence intervals (CI) were determined through multivariable logistic regression analysis to evaluate associations between cannulation techniques and outcomes. Directed acyclic graphs (DAGs) were created to investigate potential causal relationships between variables in the model and to control for confounding factors. Logistic regression model assumptions were examined, and potential confounding correlations were evaluated using both Spearman’s correlation and linear regression with an analysis of the variance inflation factor (VIF). Potential outliers were identified using Mahalanobis distance. Backward stepwise regression was employed to remove variables with *α* > 0.1. Age and sex were predetermined for inclusion in all models. IBM SPSS Statistics version 29.0.0 (241) (IBM Corp, Armonk, NY, USA) was used for statistical analysis. RStudio version 2024.4.2.764 was used to create the graphical content (RStudio: Integrated Development Environment for R. Posit Software, PBC, Boston, MA).

### Ethics

This study was approved by the Swedish Ethical Review Authority (DNR: 2023-00398-01).

## Results

As shown in Fig. 1, 4547 ERCP procedures were performed using the PCS technique, while the TPS technique was used in 3273 procedures. A control group of 39,666 ERCP procedures with regular sphincterotomy was established. Baseline characteristics are detailed in Table 1. The TPS group had a higher percentage of ASA 3–4 scores. Both PCS and TPS, particularly the PCS group, had a higher proportion of malignancy and jaundice as indications for ERCP. The percentage of intraprocedural complications was higher in both the PCS and TPS groups compared to the control group. In addition, the mean procedure time was 24.4 min for PCS compared to 28.6 min for TPS (Table 2).

**Table 1:** Baseline characteristics

Total *n* = 47,486	PCS *n* = 4547 (9.6%)	TPS *n* = 3273 (6.9%)	Control *n* = 39,666 (83.5%)
Sex, *n* (%)			
Female	2370 (52.1)	1740 (53.2)	20,889 (52.7)
Missing	1 (0.0)	2 (0.1)	10 (0.0)
Age, mean (SD), years	70.5 (15.1)	70.1 (15.7)	70.0 (16.2)
Missing	9 (0.2)	7 (0.2)	86 (0.2)
ASA, *n* (%)			
1–2	2848 (62.6)	1910 (58.4)	24,717 (62.3)
3–4	1699 (37.4)	1363 (41.6)	14,949 (37.7)
Missing	0	0	0
Indication, *n* (%)			
CBD stone	1565 (34.4)	1129 (34.5)	17,529 (44.2)
Malign./jaundice	2130 (46.8)	1369 (41.8)	13,405 (33.8)
Cholangitis	318 (7.0)	235 (7.2)	3659 (9.2)
Pancreatitis	169 (3.7)	208 (6.4)	1424 (3.6)
Missing	0	0	0
Setting, *n* (%)			
Acute	3486 (76.7)	2368 (72.3)	28,380 (71.5)
Elective	1061 (23.3)	905 (27.7)	11,286 (28.5)
Missing	0	0	0

*PCS* precut sphincterotomy, *TPS* transpancreatic sphincterotomy, *SD* standard deviation, *ASA* American Society of Anesthesiologists classification, *CBD* common bile duct

**Table 2:** Procedural characteristics

Total *n* = 47,486	PCS *n* = 4547 (9.6%)	TPS *n* = 3273 (6.9%)	Control *n* = 39,666 (83.5%)
Procedure time, mean (SD), minutes	48.0 (24.4)	50.7 (28.6)	34.9 (23.2)
Intraprocedural complication*, n (%)	231 (5.1)	144 (4.4)	969 (2.4)

*PCS* precut sphincterotomy, *TPS* transpancreatic sphincterotomy, *SD* standard deviation

*Occurring during the procedure and recorded by the endoscopist after the procedure. Not to be confounded by 30-day adverse events

**Fig. 2:**
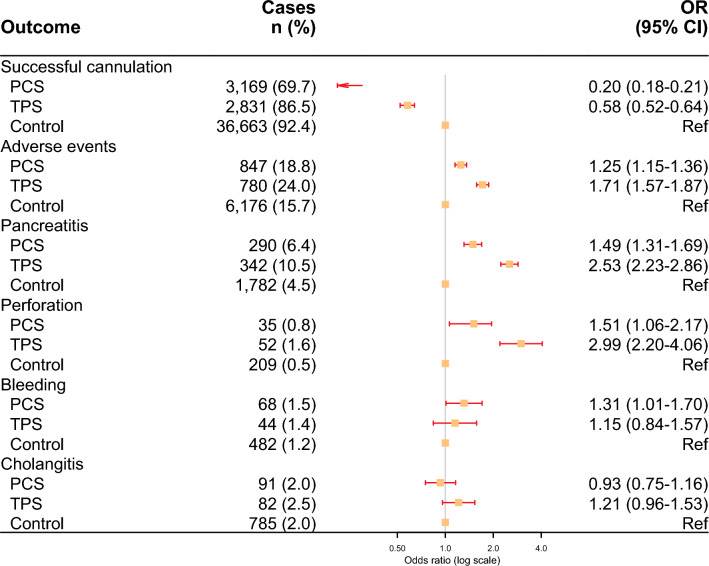
Odds ratio plot comparing PCS, TPS, and control for primary outcomes and specific adverse events. Details of the regression models are provided in the Supplemental Tables. *PCS* precut sphincterotomy, *TPS* transpancreatic sphincterotomy, *CI* confidence interval

TPS had a higher successful cannulation rate of 86.5% compared to 69.7% for PCS. This corresponded to an odds ratio of 0.58 (95% CI 0.52–0.64) for the TPS group and 0.20 (95% CI 0.18–0.21) for the PCS group compared to the control group rate of 92.4% (Fig. 2). Model adjustment and associations are shown in Supplemental Table 1. Negative associations with cannulation were found for age 63–73 years, age 74–82 years, ASA 3–4, and type 1 duodenal diverticulum. Indications including common bile duct stone, cholangitis, and pancreatitis had higher cannulation success in the regression model compared to malignancy/jaundice, as did an acute setting (Supplemental Table 1). The TPS group had the highest rate of adverse events, at 24.0%, followed by the PCS group at 18.8% and the control group at 15.7% (Fig. 2). This corresponded to an odds ratio of 1.71 for TPS (95% CI 1.57–1.87) and 1.25 for PCS (95% CI 1.15–1.36) with individually separated confidence intervals compared with the control group (Fig. 2). The adverse event model is detailed in Supplemental Table 1. Negative associations were observed with older age, ASA 3–4, stone, and pancreatitis as an indication. In contrast, an acute setting was associated with a higher incidence of adverse events. Specific outcomes were analyzed in separate models, which are shown in Supplemental Tables 2, 3. These demonstrated an incidence of pancreatitis of 6.4% for PCS (OR 1.49; 95% CI 1.31–1.69), 10.5% for TPS (OR 2.53; 95% CI 2.23–2.86), and 4.5% for the control group. The incidence of perforation was also different among the groups with 0.8% for PCS (OR 1.51; 95% CI 1.06–2.17), 1.6% for TPS (OR 2.99; 95% CI 2.20–4.06), and 0.5% for the control group (Supplemental Table 2).

## Discussion

Previous studies comparing the PCS and TPS techniques have been limited to smaller case series, all of which have been included in a systematic review [14]. Our study presents the largest cohort to date, demonstrating a significant advantage in success rate for the TPS technique over PCS. However, this advantage is accompanied by a heightened risk of adverse events that has not been previously demonstrated. Nevertheless, it is important to note that these techniques are not strictly comparable. The TPS technique requires the inadvertent insertion of a guidewire into the pancreatic duct. If this is not achieved, a number of options are available, including continuing with the standard cannulation technique, attempting the PCS technique, aborting the procedure, using endoscopic ultrasound (EUS) for cannulation, or opting for percutaneous biliary drainage (PTBD).

The higher successful cannulation rate for the TPS technique compared to PCS is consistent with most previous studies comparing these techniques. The reported cannulation rate of 86% for TPS is also in line with the results of previous studies [1, 6, 8–10, 14]. However, other studies have also described similar cannulation rates between these two techniques [7, 11]. PCS and the use of a needle knife is a more technically demanding technique than TPS and requires the endoscopist to be more experienced with ERCP. Our cohort represents procedures performed by endoscopists from different types of hospitals and not only from specialized centers, which may be represented in smaller series. This may explain why the PCS cannulation rate in smaller series is generally higher than the 70% observed in our study. Our results also show that the PCS technique was more frequently used when the indication for ERCP was malignancy. This setting may result in a more difficult biliary access in general, which may be an additional explanation for the lower success rate of the PCS technique.

Most studies and systematic review by Lyu et al. have reported similar rates of adverse events, including PEP, when comparing the two techniques [14]. We found a higher rate (24%) of overall adverse events with the use of TPS compared to PCS (18.8%). Our adverse event rates for these techniques are higher than those reported in previous studies [1, 6–11]. However, our PEP rate of 10% for TPS and 6.4% for PCS are more in line with what has been previously shown [11, 14]. Nevertheless, no study has shown a doubled risk of PEP with TPS, although Halttunen et al. reported a trend toward a higher risk of PEP with TPS [10]. Regarding the outcome of perforation, it is uncommon to find comparable perforation rates in smaller series due to the low incidence. Our findings indicate an increased perforation rate for both techniques, with TPS exhibiting the highest rate. Additionally, we observed a slightly higher bleeding rate for PCS compared to the control group, confirming previous findings [6, 13, 14].

There are several possible explanations and limitations to our findings. One drawback of our study is the lack of adjustment for PEP prevention measures, such as rectal NSAID use and pancreatic stent placement. We speculate that the use of pancreatic stents was less common in the early years of our cohort (due to routine and guideline statements), possibly leading to a lower rate of PEP for TPS in more recent years. As the body of measures to reduce adverse events (especially PEP) during ERCP has grown, further research focusing on more recent years may show less difference in adverse events comparing these techniques.

The TPS technique has also evolved over the years. The wire-guided TPS technique is now standard, but TPS was originally performed without a guidewire, using only a sphincterotome and contrast injection. Chan et al. also discussed how aggressive TPS can be performed with a small cut toward the bile duct or a larger, more aggressive sphincterotomy to visualize the distal bile duct [9]. In our cohort, we cannot definitively determine how the TPS technique was applied. We were also unable to adjust for the use of the DGW technique prior to TPS. It is likely that DGW was used due to current guidelines [3], but some reports have suggested that DGW itself may increase the risk of PEP [15, 18, 19]. Therefore, the lack of knowledge about the use of DGW is a substantial limitation of our study. Additionally, the number of regular attempts at cannulation before using DGW and the number of attempts with DGW before switching to TPS are unknown. The longer average procedure time we found for TPS may account for more repeated cannulation attempts, which could increase the risk of PEP.

One could argue that, given our result with a higher incidence of adverse events associated with TPS, other options such as PTBD or EUS should be considered. EUS biliary drainage has an estimated adverse event rate of 14% [20], and PTBD has a rate of approximately 12% [21], both much lower than our adverse event rate of 24% for TPS. Studies comparing EUS or PTBD with ERCP have predominantly been in the setting of malignant obstructive jaundice, with comparable risk of adverse events, although ERCP has a higher risk of PEP [22–25]. However, ERCP has the advantage of a shorter hospital stay and is more cost-effective [26]. Also, these other techniques may not be readily available, and if unintentional pancreatic duct cannulation occurs during an attempted ERCP, some of the increased risk of PEP may already have been induced. Conversely, early use of PCS is known to reduce PEP, likely due to less papillary manipulation [1]. Wang et al. discussed whether the same could be applied to TPS, suggesting that performing an “early TPS” could reduce adverse events [11], a topic to be investigated in future studies.

In conclusion, the use of TPS results in a higher cannulation success rate compared to PCS. This is accompanied by an increased risk of adverse events, mainly manifested by increased rates of PEP and perforation. Nevertheless, we maintain that the TPS technique should still be considered as a valuable tool in the arsenal of cannulation strategies.

## Supplementary Information

Below is the link to the electronic supplementary material.

Supplementary file1 (PDF 62 kb)

Supplementary file2 (PDF 60 kb)

Supplementary file3 (PDF 60 kb)
