# Supplementary material for: Difficult cannulation during endoscopic retrograde cholangiopancreatography—needle-knife precut versus transpancreatic sphincterotomy on the basis of successful cannulation and adverse events
Source: Surg Endosc. 2024 Dec 29;39(2):1200–6. doi: 10.1007/s00464-024-11429-y (PMC11794349; doi:10.1007/s00464-024-11429-y)
Supplement: Supplementary file 1 — Supplementary file1 (PDF 62 kb) [file 464_2024_11429_MOESM1_ESM.pdf]

Variable	Successful cannulation: Cases (%)	Successful cannulation: OR (95% CI)	Adverse events: Cases (%)	Adverse events: OR (95% CI)
technique				
PCS	3,169 (69.7)	0.19 (0.18-0.20)<br>0.20 (0.18-0.21)	847 (18.8)	1.24 (1.15-1.35)<br>1.25 (1.15-1.36)
TPS	2,831 (86.5)	0.52 (0.47-0.58)<br>0.58 (0.52-0.64)	780 (24)	1.70 (1.56-1.85)<br>1.71 (1.57-1.87)
Control	36,663 (92.4)	Ref	6,176 (15.7)	Ref
sex				
female	22,546 (90.2)	1.08 (1.02-1.15)<br>1.03 (0.97-1.10)	4,159 (16.7)	1.03 (0.98-1.08)<br>1.04 (0.99-1.09)
male	20,107 (89.5)	Ref	3,640 (16.3)	Ref
age				
>83	9,853 (90.8)	0.93 (0.85-1.02)<br>0.99 (0.89-1.09)	1,401 (13)	0.53 (0.50-0.57)<br>0.79 (0.74-0.85)
74-82	11,170 (88.8)	0.75 (0.69-0.82)<br>0.85 (0.77-0.93)	1,812 (14.5)	0.60 (0.57-0.65)<br>0.70 (0.66-0.76)
63-73	10,559 (88.5)	0.72 (0.67-0.79)<br>0.86 (0.78-0.94)	1,966 (16.6)	0.71 (0.66-0.76)<br>0.64 (0.59-0.69)
<=62	10,987 (91.4)	Ref	2,606 (21.9)	Ref
ASA				
3-4	15,927 (88.4)	0.78 (0.74-0.83)<br>0.81 (0.76-0.86)	2,660 (14.9)	0.82 (0.78-0.86)<br>0.95 (0.89-1.00)
1-2	26,736 (90.7)	Ref	5,143 (17.6)	Ref
indication				
stone	18,856 (93.2)	2.52 (2.35-2.70)<br>2.35 (2.18-2.52)	2,885 (14.3)	0.91 (0.86-0.96)<br>0.95 (0.90-1.00)
cholangitis	4,015 (95.3)	3.73 (3.21-4.33)<br>3.73 (3.20-4.36)	602 (14.4)	0.91 (0.83-1.00)
pancreatitis	1,589 (88.2)	1.37 (1.18-1.59)<br>1.27 (1.09-1.48)	221 (12.4)	0.77 (0.66-0.89)<br>0.72 (0.62-0.83)
J/M	14,291 (84.5)	Ref	2,616 (15.6)	Ref
setting				
acute	30,748 (89.8)	0.99 (0.93-1.06)<br>1.11 (1.03-1.19)	5,779 (17)	1.13 (1.07-1.19)<br>1.39 (1.31-1.47)
elective	11,915 (89.9)	Ref	2,024 (15.4)	Ref
type 1 diverticula				
yes	1,504 (82)	0.50 (0.44-0.56)<br>0.36 (0.32-0.42)		
no	41,159 (90.2)	Ref		
type 2 diverticula				
yes			640 (15.9)	0.95 (0.87-1.04)
no			7,163 (16.6)	Ref

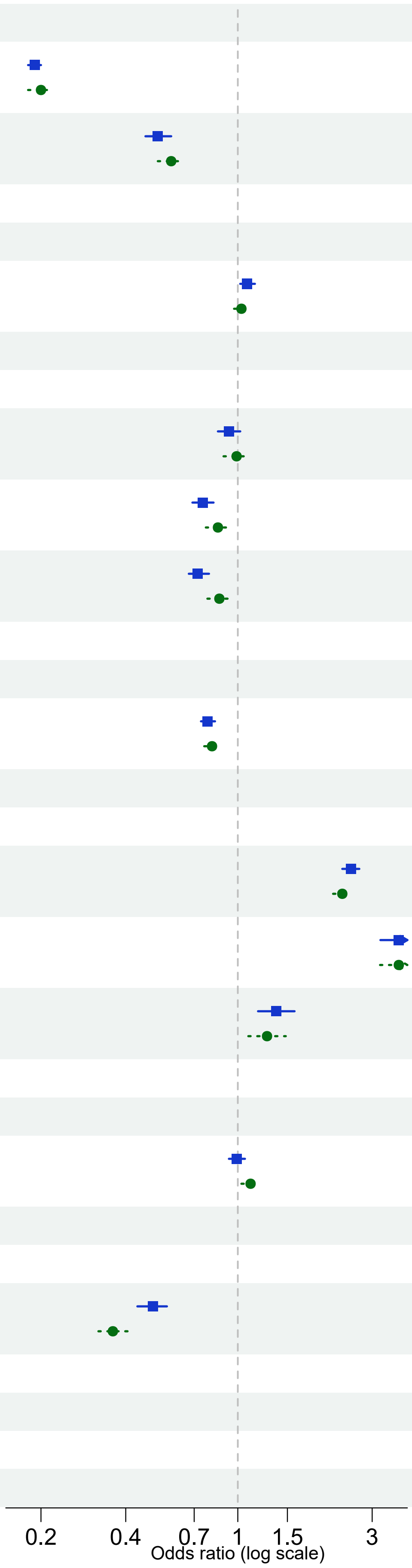

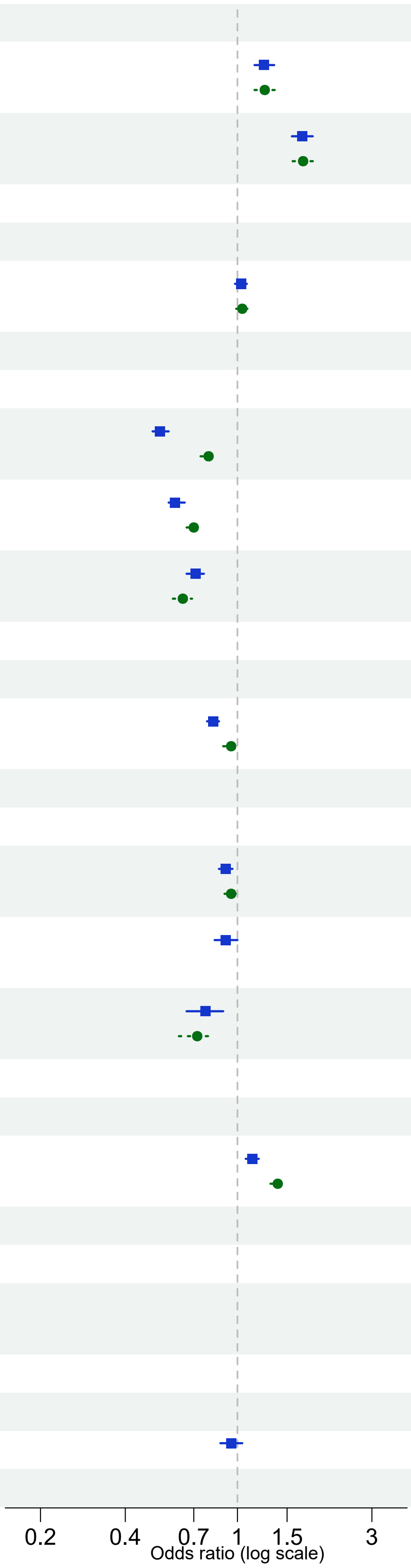

Univariable · Multivariable

J/M. jaundice/malignancy
Model 1: χ2(df = 13, n = 47,384) = 2758.88, P < .001, Nagelkerke R2 = 11.7%, classification = 89.8%
Model 2: χ2(df = 11, n = 47,060) = 1376.06, P < .001, Nagelkerke R2 = 5.0%, classification = 83.5%
