# Supplementary material for: Difficult cannulation during endoscopic retrograde cholangiopancreatography—needle-knife precut versus transpancreatic sphincterotomy on the basis of successful cannulation and adverse events
Source: Surg Endosc. 2024 Dec 29;39(2):1200–6. doi: 10.1007/s00464-024-11429-y (PMC11794349; doi:10.1007/s00464-024-11429-y)
Supplement: Supplementary file 2 — Supplementary file2 (PDF 60 kb) [file 464_2024_11429_MOESM2_ESM.pdf]

	Pancreatitis		Perforation	
**Variable**	**Cases (%)**	**OR (95% CI)**	**Cases (%)**	**OR (95% CI)**
technique				
PCS	290 (6.4)	1.45 (1.28-1.65)<br>1.49 (1.31-1.69)	35 (0.8)	1.47 (1.02-2.10)<br>1.51 (1.06-2.17)
TPS	342 (10.5)	2.48 (2.20-2.80)<br>2.53 (2.23-2.86)	52 (1.6)	3.05 (2.24-4.14)<br>2.99 (2.20-4.06)
Control	1,782 (4.5)	Ref	209 (0.5)	Ref
sex				
female	1,363 (5.5)	1.18 (1.08-1.28)<br>1.15 (1.06-1.26)	164 (0.7)	1.12 (0.89-1.41)<br>1.11 (0.88-1.40)
male	1,050 (4.7)	Ref	132 (0.6)	Ref
age				
>83	359 (3.3)	0.40 (0.35-0.45)<br>0.49 (0.43-0.56)	46 (0.4)	0.51 (0.36-0.72)<br>0.68 (0.47-0.98)
74-82	523 (4.2)	0.50 (0.45-0.56)<br>0.56 (0.50-0.63)	81 (0.6)	0.77 (0.57-1.03)<br>0.96 (0.71-1.31)
63-73	580 (4.9)	0.59 (0.53-0.66)<br>0.63 (0.57-0.71)	69 (0.6)	0.69 (0.51-0.94)<br>0.82 (0.60-1.12)
<=62	948 (8.0)	Ref	100 (0.8)	Ref
ASA				
3-4	681 (3.8)	0.63 (0.57-0.69)<br>0.79 (0.71-0.87)	103 (0.6)	0.87 (0.69-1.11)
1-2	1,733 (5.9)	Ref	193 (0.7)	Ref
indication				
stone	977 (4.9)	0.95 (0.86-1.04)<br>0.89 (0.80-0.98)	112 (0.6)	1.23 (0.92-1.65)
cholangitis	142 (3.4)	0.65 (0.55-0.78)<br>0.84 (0.70-1.01)	23 (0.6)	1.22 (0.76-1.94)
pancreatitis	75 (4.2)	0.82 (0.64-1.04)<br>0.73 (0.57-0.93)	8 (0.4)	0.99 (0.48-2.06)
J/M	858 (5.1)	Ref	76 (0.5)	Ref
setting				
acute	1,530 (4.5)	0.65 (0.60-0.71)<br>0.74 (0.68-0.81)	195 (0.6)	0.75 (0.59-0.95)
elective	884 (6.7)	Ref	101 (0.8)	Ref
type 2 diverticula				
yes	171 (4.3)	0.81 (0.69-0.95)	31 (0.8)	1.26 (0.87-1.83)<br>1.41 (0.96-2.06)
no	2,243 (5.2)	Ref	265 (0.6)	Ref

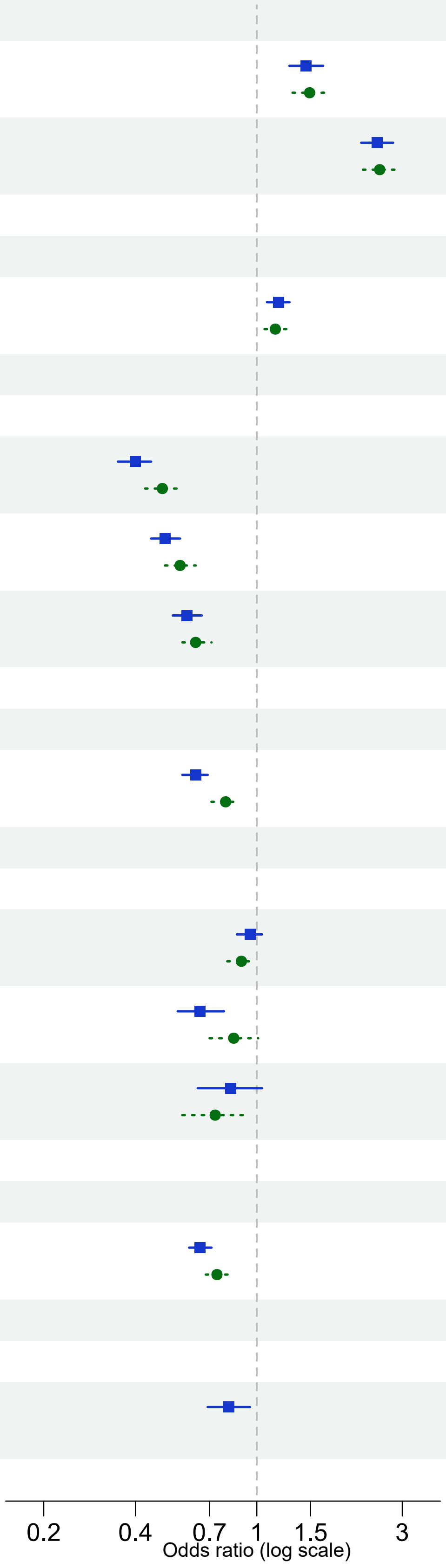

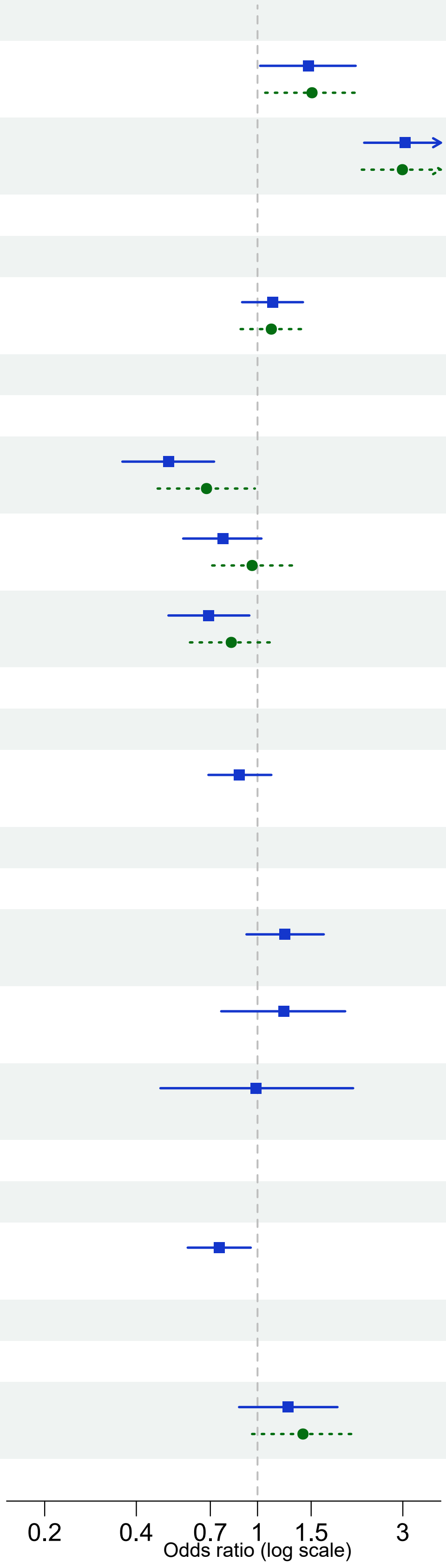

J/M. jaundice/malignancy
Model 1: χ2(df = 12, n = 47,060) = 607.27, P < .001,
Nagelkerke R2 = 3.9%, classification = 94.9%
Model 2: χ2(df = 8, n = 47,060) = 122.46, P < .001,
Nagelkerke R2 = 3.5%, classification = 99.4%

Univariable Multivariable
