# Supplementary material for: Difficult cannulation during endoscopic retrograde cholangiopancreatography—needle-knife precut versus transpancreatic sphincterotomy on the basis of successful cannulation and adverse events
Source: Surg Endosc. 2024 Dec 29;39(2):1200–6. doi: 10.1007/s00464-024-11429-y (PMC11794349; doi:10.1007/s00464-024-11429-y)
Supplement: Supplementary file 3 — Supplementary file3 (PDF 60 kb) [file 464_2024_11429_MOESM3_ESM.pdf]

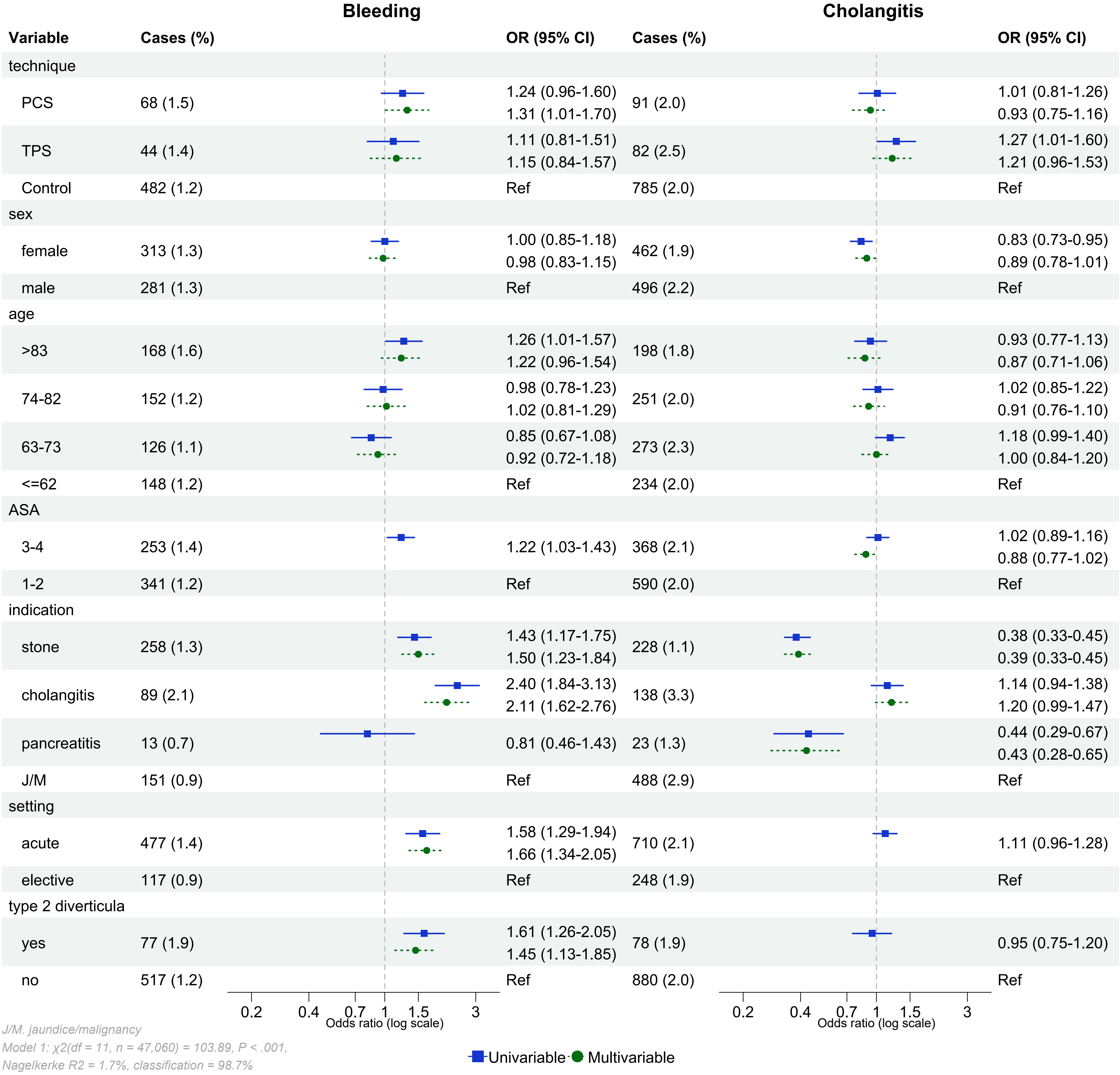

Variable	Bleeding: Cases (%)	Bleeding: OR (95% CI)	Cholangitis: Cases (%)	Cholangitis: OR (95% CI)
technique				
PCS	68 (1.5)	1.24 (0.96-1.60)<br>1.31 (1.01-1.70)	91 (2.0)	1.01 (0.81-1.26)<br>0.93 (0.75-1.16)
TPS	44 (1.4)	1.11 (0.81-1.51)<br>1.15 (0.84-1.57)	82 (2.5)	1.27 (1.01-1.60)<br>1.21 (0.96-1.53)
Control	482 (1.2)	Ref	785 (2.0)	Ref
sex				
female	313 (1.3)	1.00 (0.85-1.18)<br>0.98 (0.83-1.15)	462 (1.9)	0.83 (0.73-0.95)<br>0.89 (0.78-1.01)
male	281 (1.3)	Ref	496 (2.2)	Ref
age				
>83	168 (1.6)	1.26 (1.01-1.57)<br>1.22 (0.96-1.54)	198 (1.8)	0.93 (0.77-1.13)<br>0.87 (0.71-1.06)
74-82	152 (1.2)	0.98 (0.78-1.23)<br>1.02 (0.81-1.29)	251 (2.0)	1.02 (0.85-1.22)<br>0.91 (0.76-1.10)
63-73	126 (1.1)	0.85 (0.67-1.08)<br>0.92 (0.72-1.18)	273 (2.3)	1.18 (0.99-1.40)<br>1.00 (0.84-1.20)
<=62	148 (1.2)	Ref	234 (2.0)	Ref
ASA				
3-4	253 (1.4)	1.22 (1.03-1.43)	368 (2.1)	1.02 (0.89-1.16)<br>0.88 (0.77-1.02)
1-2	341 (1.2)	Ref	590 (2.0)	Ref
indication				
stone	258 (1.3)	1.43 (1.17-1.75)<br>1.50 (1.23-1.84)	228 (1.1)	0.38 (0.33-0.45)<br>0.39 (0.33-0.45)
cholangitis	89 (2.1)	2.40 (1.84-3.13)<br>2.11 (1.62-2.76)	138 (3.3)	1.14 (0.94-1.38)<br>1.20 (0.99-1.47)
pancreatitis	13 (0.7)	0.81 (0.46-1.43)	23 (1.3)	0.44 (0.29-0.67)<br>0.43 (0.28-0.65)
J/M	151 (0.9)	Ref	488 (2.9)	Ref
setting				
acute	477 (1.4)	1.58 (1.29-1.94)<br>1.66 (1.34-2.05)	710 (2.1)	1.11 (0.96-1.28)
elective	117 (0.9)	Ref	248 (1.9)	Ref
type 2 diverticula				
yes	77 (1.9)	1.61 (1.26-2.05)<br>1.45 (1.13-1.85)	78 (1.9)	0.95 (0.75-1.20)
no	517 (1.2)	Ref	880 (2.0)	Ref

J/M. jaundice/malignancy
Model 1: χ2(df = 11, n = 47,060) = 103.89, P < .001, Nagelkerke R2 = 1.7%, classification = 98.7%
Model 2: χ2(df = 11, n = 47,060) = 201.65, P < .001, Nagelkerke R2 = 2.4%, classification = 98.0%
